# Saudi SCD patients’ symptoms and quality of life relative to the number of ED visits

**DOI:** 10.1186/s12873-016-0096-z

**Published:** 2016-08-20

**Authors:** Anwar E. Ahmed, Ahmed S. Alaskar, Donna K. McClish, Yosra Z. Ali, Mohammed H. Aldughither, Ahmad M. Al-Suliman, Hafiz M. Malhan

**Affiliations:** 1 King Saud bin Abdulaziz University for Health Sciences, College of Public Health and Health Informatics, MC 2350, P.O.Box 22490, Riyadh, 11426, KSA Saudi Arabia; 2 King Abdullah International Medical Research Center, Riyadh, Saudi Arabia; 3 King Abdulaziz Medical City, Riyadh, Saudi Arabia; 4 Department of Biostatistics, Virginia Commonwealth University, Richmond, VA USA; 5 National Anti-Corruption Commission, Riyadh, Saudi Arabia; 6 King Fahad Hospital, Hofuf, Saudi Arabia; 7 King Fahad Central Hospital, Jazan, Saudi Arabia

**Keywords:** Sickle cell disease, Emergency department visits, Pain, Inherited anemia, Quality of life

## Abstract

**Background:**

Individuals living with sickle cell disease (SCD) have significantly increased emergency department (ED) use compared to the general population. In Saudi Arabia, health care is free for all individuals and therefore has no bearing on increased ED visits. However, little is known about the relationship between quality of life (QoL) and frequency of acute care utilization in this patient population.

**Methods:**

A cross-sectional study was conducted on 366 patients with SCD who attended the outpatient department at King Fahad Hospital, Hofuf, Saudi Arabia. Data were collected through self-administered surveys, which included: demographics, SCD-related ED visits, clinical issues, and QoL levels. We assessed the ED use by asking for the number of SCD-related ED visits within a 6-month period.

**Results:**

The self-report survey of ED visits was completed by 308 SCD patients. The median number of SCD-related ED visits within a 6-month time period (IQR) was four (2-7 visits). According to the unadjusted negative binomial model, the rate of SCD-related ED visits increased by (46, 39.3, 40, and 53.5 %) for patients with fever, skin redness with itching, swelling, and blood transfusion, respectively. Poor QoL tends to increase the rate of SCD-related ED visits. Well education and poor general health positively influenced the rate of SCD-related ED visits. Well education tends to increase the rate of SCD-related ED visits by 50.2 %. The rate of SCD-related ED visits decreased by 1.4 % for every point increase in general health.

**Conclusion:**

Saudi patients with sickle cell disease reported a wide range of SCD-related ED visits. It was estimated that six of 10 SCD patients had at least three ED visits within a 6-month period. Well education and poor general health resulted in an increase in the rate of SCD-related ED visits.

## Background

Sickle cell disease (SCD) is comprised of a group of inherited red blood cell disorders characterized by the presence of abnormal hemoglobin. SCD is present throughout Saudi Arabia, mostly in the eastern and southern provinces. Information about the prevalence of SCD in Saudi Arabia is not well established, but studies to date have reported that SCD is a relatively common genetic disorder in this part of the world [1–3].

The clinical manifestations of SCD often lead the patient to visit the emergency department [4–8]. The most common manifestation of SCD is vaso-occlusive crisis, which can cause a multitude of major organ complications as well as result in recurrent painful episodes [7, 9–14]. This increases the risks for infections and other health complications that require emergency intervention [15–17]. In particular, high rates of ED utilization have been reported in single and multi-institutional studies [4, 6, 7, 11, 18–24]. The predictors of frequent ED utilization are not fully understood [20], but the following variables have been found to be associated with higher ED and hospital utilization: (1) the more severe disease and disease-related complications, (2) the need for pain crisis management [25], (3) higher white blood cell counts, (4) lower hemoglobin levels, (5) asthma, (6) being publically insured, (7) being 18 to 30 years old, and (8) the inability to obtain follow-up appointments with hematology. Within these presentations, pain is the most common complication of SCD requiring ED visits and hospitalization.

Our patients with SCD receive healthcare by routinely visiting primary care physicians for health checkups, managing pain, and urine/blood tests. Many of these patients do not have an assigned hematologist to provide counseling or advice on pain management. However, primary care physicians may make referrals to the Hematology Outpatient Clinic where necessary. Although healthcare is free to all individuals in Saudi Arabia, it is unknown whether all affected patients have access to a disease specialist.

Because the health system in Saudi Arabia provides free access to the Saudi population, patients with SCD have access not only to specialized health care but also to home pain medications. This includes over-the-counter pain medications such as Advil, Tylenol, etc. to control mild pain at home. Saudi SCD patients may have opioid pain medication available at home upon doctor prescription. At the ED, patients may request prescriptions for stronger medicines such as opioid medications, including morphine, tramadol, hydrocodone, methadone, fentanyl, codeine, and meperidine. However, as is the case with any chronic disease, patient compliance continues to be an important issue.

Self-report measurements of pain and wellbeing as measured by the 36-Item Short Form Health Survey (SF-36) may predict frequent ED visits in patients with SCD. Increased life expectancy due to recent medical advances has increased the need to understand more fully the health-related quality of life (HRQoL) [26] in patients with SCD [2]. Previous research has indicated that patients with SCD experience a lower HRQoL [1, 2, 25, 27–33] compared to the general adult population. A number of disease-related factors have been found to affect HRQoL in patients with SCD. These can develop into life-threatening complications as well as extensive organ damage, reducing both quality of life (QoL) and life expectancy. A clinically important reduction in the HRQoL and the presence of other prognostic factors can further contribute to determining the reasons for ED visits [34].

The presence of complications and risk factors may also raise the risk level of ED visits in patients with SCD, such as swelling in the hands or feet, fever, blood transfusion, and family history of anemia. The current investigation studied a sample of SCD patients from Saudi Arabia to assess the frequency of ED use and its relation to patients’ symptoms and QoL.

## Methods

This was a cross-sectional study of patients with SCD who attended the outpatient clinic of a local hospital in Saudi Arabia from October 1, 2014 to February 29, 2015. Participants of the study were adult patients with SCD who were treated in the Inherited Blood Disorder Center, King Fahad Hospital (KFH) - Hofuf, Eastern Region, Saudi Arabia. KFH - Hofuf is the main hospital in the Al-Ahsa region in eastern Saudi Arabia. Serving about one and a half million people from the general population of Al-Ahsa, it receives cases from Al-Ahsa region health centers and government and private hospitals.

The study population was composed of SCD patients who were attending for a follow-up visit to the Hematology Outpatient Clinic. This study was approved by the Ministry of Health, Kingdom of Saudi Arabia (IRB Log No. 15-247E) and King Abdullah International Medical Research Center, Research Protocol - RC12/127/R. The study conducted an anonymous survey of patients with SCD, and no identifiable data were collected from the patients. Only patients who agreed to participate in the study were asked to complete and return the survey.

Patients’ characteristics—such as age, gender, frequency of ED visits, marital status, employment, and education level—were collected. The following clinical characteristics were assessed: duration of illness, obesity, chronic disease other than SCD, and whether the patient had a history of inherited anemia and a history of blood transfusion. Complications and symptoms of SCD within the past 6 months were assessed through self-report, such as fever and swelling of the hands or feet. Since skin redness with itching can be a result of infections or side effects from certain SCD medications, and skin redness with itching can be a potential indicator of poor QoL, the study collected data on this characteristic as well. We evaluated the frequency of ED visits by asking for the number of SCD-related ED visits within the past six months. A validated and Arabic-translated RAND-36 measure of health-related quality of life (HRQoL) questionnaire [35] was also administered to the study participants. Patients rated their HRQoL in terms of their feelings and perceptions with regard to eight different components: physical function; physical role health; emotional role functions; vitality; emotional wellbeing; social function; bodily pain; and general health perceptions. Each scale scored from 0 = least favorable health state to 100 = most favorable health state. The Arabic version of RAND-36 HRQoL was found to be reliable [36]. More details of the study are discussed by Ahmed et al. [36]. A total of 450 surveys were distributed (81.3 % response rate), and 366 were returned.

### Statistical analysis

The data analyses were conducted using IBM SPSS Statistics (20; SPSS, Chicago, IL). Descriptive statistics were used to describe continuous and categorical variables (Table 1). We used negative binomial regression model to identify the risk factors associated with the high rate of SCD-related ED visits (Table 2). We assessed the adequacy of multivariate regression model by comparing the deviance and the log likelihood for negative binomial and Poisson regression models. In all analyses, *P* < 0.05 was considered significant.

**Table 1:** Participant characteristics

Characteristics	*Levels*	Number	Percent
Age/year	*Mean ± SD*	29.0 ± 10.5	
Number of SCD-related ED visits	*Median(IQR)*	4(2–7)	
Female gender	*No*	273	74.6
	*Yes*	93	25.4
University	*No*	269	74.5
	*Yes*	92	25.5
Married	*No*	164	45.6
	*Yes*	196	54.4
Unemployed	*No*	124	36.6
	*Yes*	215	63.4
Regular exercise	*No*	232	64.3
	*Yes*	129	35.7
Obese	*No*	296	90.8
	*Yes*	30	9.2
Fever	*No*	186	51.4
	*Yes*	176	48.6
Skin redness with itching	*No*	242	67.0
	*Yes*	119	33.0
Swelling	*No*	219	60.5
	*Yes*	143	39.5
Blood transfusion	*No*	97	26.6
	*Yes*	267	73.4
Family history of anemia	*No*	28	7.7
	*Yes*	335	92.3
Family support	*No*	39	11.0
	*Yes*	317	89.0
Chronic disease other than SCD	*No*	303	84.4
	*Yes*	56	15.6

**Table 2:** Factors associated with the rate of SCD-related ED visits

	Unadjusted model					Multivariate adjusted model				
				95 % Wald CI for RR					95 % Wald CI for RR	
	B	P	RR	Lower	Upper	B	P	RR	Lower	Upper
Male gender	.285	.050^a^	1.329	1.001	1.768	.137	.513	1.146	.762	1.725
University degree	.147	.298	1.159	.878	1.529	.407	.026^a^	1.502	1.050	2.149
Married	.345	.006^a^	1.413	1.106	1.804	−.153	.456	.858	.574	1.283
Unemployed	−.294	.024^a^	.745	.577	.962	−.308	.114	.735	.502	1.077
Regular exercise	−.161	.212	.851	.660	1.096	−.259	.148	.772	.544	1.096
Obese	.182	.434	1.199	.761	1.889	.453	.136	1.573	.868	2.850
Fever	.378	.002^a^	1.460	1.146	1.860	.032	.852	1.032	.741	1.438
Skin redness with itching	.331	.011^a^	1.393	1.080	1.796	.002	.993	1.002	.693	1.447
Swelling	.337	.007^a^	1.400	1.094	1.792	.134	.464	1.143	.799	1.635
Blood transfusion	.429	.002^a^	1.535	1.165	2.023	.152	.407	1.164	.813	1.667
Family history of anemia	.294	.227	1.342	.833	2.161	−.012	.970	.988	.522	1.869
Family support	.337	.097	1.401	.941	2.085	.207	.408	1.230	.753	2.012
Chronic disease other than SCD	−.088	.617	.916	.650	1.291	−.248	.340	.781	.469	1.299
Age	.013	.034^a^	1.013	1.001	1.025	.001	.892	1.001	.982	1.022
Physical function	−.007	.010^a^	.993	.988	.998	.002	.721	1.002	.993	1.010
Physical role functions	−.006	.001^a^	.994	.991	.998	−.002	.601	.998	.993	1.004
Emotional role functions	−.004	.007^a^	.996	.993	.999	−.003	.153	.997	.993	1.001
Vitality	−.012	.001^a^	.988	.981	.995	−.002	.708	.998	.987	1.009
Emotional well-being	−.005	.113	.995	.988	1.001	.002	.680	1.002	.991	1.013
Social function	−.009	.001^a^	.991	.986	.996	−.002	.687	.998	.989	1.007
Bodily pain	−.008	.001^a^	.992	.988	.997	−.004	.281	.996	.988	1.004
General health	−.018	.001^a^	.982	.975	.989	−.014	.018^a^	.986	.975	.998

^a^Significant at α = 0.05

## Results

Of 366 patients, 58 (15.8 %) chose not to report their SCD-related ED utilization within the past six months, thus the self-report of SCD-related ED visits was completed by 308 SCD patients. Of the 308 SCD patients, only 21 (6.8 %) reported no SCD-related ED visits, 28 (9.1 %) reported one SCD-related ED visit, and 61 (19.8 %) reported two SCD-related ED visits. The majority of our samples, 198 (64.3 %), reported three or more SCD-related ED visits over the past 6 months (95 % confidence limit: 58.7–69.6 %). The median number of SCD-related ED visits was four (IQR 2–7) with ranges between 0 and 87 visits.

The SCD patients’ average age was 29 (SD = 10.5) years, with 74.6 % being male and 35.7 % reporting regular exercise. Table 2 shows the factors associated with the rate of SCD-related ED visits. Unadjusted analyses show that the rate of SCD-related ED visits was higher for the male gender (RR: 1.329 [95 % CI: 1.001–1.768, *P* = 0.050]), married (RR: 1.413 [95 % CI: 1.106–1.804, *P* = 0.006]), fever (RR: 1.460 [95 % CI: 1.146–1.860, *P* = 0.002]), skin redness with itching (RR: 1.393 [95 % CI: 1.080–1.796, *P* = 0.011]), swelling (RR: 1.4 [95 % CI: 1.094–1.792, *P* = 0.007]), blood transfusion (RR: 1.535 [95 % CI: 1.165–2.023, *P* = 0.002]), and older age (RR: 1.013 [95 % CI: 1.001–1.025, *P* = 0.034]).

The rate of SCD-related ED visits was less for unemployed patients (RR: 0.745 [95 % CI: 0.577–0.962, *P* = 0.024]). The rate of SCD-related ED visits decreased for every point increase in physical function (RR: 0.993 [95 % CI: 0.988–0.998, *P* = 0.010]), physical role functions (RR: 0.994 [95 % CI: 0.991–0.998, *P* = 0.001]), emotional role functions (RR: 0.996 [95 % CI: 0.993–0.999, *P* = 0.007]), vitality (RR: 0.988 [95 % CI: 0.981–0.995, *P* = 0.001]), social function (RR: 0.991 [95 % CI: 0.986–0.996, *P* = 0.001]), and general health (RR: 0.982 [95 % CI: 0.975–0.989, *P* = 0.001]). The rate of SCD-related ED visits decreased for every point decrease in bodily pain (RR: 0.992 [95 % CI: 0.988–0.997, *P* = 0.001]).

Adjusted analysis in Table 2 shows the factors associated with the high rate of SCD-related ED visits. According to the multivariable negative binomial regression model, the rate of SCD-related ED visits was much higher for those with a university degree (RR: 1.502 [95 % CI: 1.050–2.149, *P* = 0.026]). The rate of SCD-related ED visits decreased for every point increase in general health (RR: 0.986 [95 % CI: 0.975–0.998, *P* = 0.018]).

The deviance value for the negative binomial regression model was near to1.0 (0.96), while for a Poisson regression model was (6.937). The log likelihood was smaller for the Poisson regression model compared to the negative binomial regression model ( −1066.150 vs. −644.880), which indicates the negative binomial regression model may be appropriate.

## Discussion

This study represents the first investigation in the entire Arabic world on SCD patients’ symptoms and QoL relative to the number of their SCD-related ED visits. We found that the frequent SCD-related ED utilization (three SCD-related ED visits or greater within a 6-month time period) was very prevalent in the Saudi population, with 64.3 % (95 % confidence limit: 58.7–69.6 %). A small number of sickle cell patients (28.9 %) reported between one and two SCD-related ED visits within a 6-month time period, while (6.8 %) reported no SCD-related ED visits. Previous studies, which were based on a daily diary of ED utilization for pain management and other related causes, reported 15–35.5 % of the patients with SCD had three or more ED visits per year [8, 37, 38]. The current investigation showed higher rates of ED utilization than what has been reported in these studies.

A retrospective exploratory chart review study found that SCD patients with multiple ED visits in the past 12 months reported significantly higher pain scores [39]. Given that the methodology of Marco CA et al. was different compared to that of the present study, our unadjusted analysis is in line with their findings that the rate of SCD-related ED visits increased because of pain. A survey with similar methodology to our study was conducted by the CDC National Center for Health Statistics, which reported pain as the most common reason for the ED visit [40]. Another prospective study reported nearly 37 % of ED use was because of painful episodes [41].

A retrospective study suggested that poorer physical HRQoL domains were significantly correlated to more healthcare utilization events [42]. Our unadjusted analyses confirmed these findings: all dimensions of HRQoL were found to be predictors of high rates of SCD-related ED visits except emotional well-being. According to our unadjusted analyses, patients with clinical symptoms such as fever, skin redness with itching, swelling, or blood transfusion tend to have high rates of SCD-related ED visits. These findings are in agreement with Yusuf HR et al. [40] and Koshy M et al. [41], which have cited fever as a reason for ED visits in patients with SCD.

In the multivariate negative binomial regression model, the rate of SCD-related ED visits (50.2 % were higher for SCD patients with a university degree. Using an increase in general health ratings by a score of 1, the rate of SCD-related ED visits decreased by 1.4 %.

Several methodological limitations could narrow our interpretations: The study was based on a cross-sectional design, and therefore provides more data about association than causation. ED utilization was based on self-reporting, not on a retrospective or prospective chart review for each visit. Our sample recruited from SCD patients who attended an outpatient clinic for a follow-up visit, which may not represent the entire SCD population. Some modifiable factors were not taken into consideration, such as having a hematologist, or struggling with financial or transportation issues. Despite these limitations, this is the first study that has reported the ED utilization in patients with SCD in the entire Arab region. While our study highlights ED utilization in SCD patients, and despite its limitations, this is the first study to highlight predictors of ED visits among the population of Saudi Arabian SCD patients. The findings that the level of educational attainment and poorer general health are independent risk factors of SCD-related ED visits can assist medical professionals in informing and educating SCD patients about disease management.

## Conclusions

Saudi patients with sickle cell disease reported a wide range of SCD-related ED visits. It was estimated that six of 10 SCD patients had at least three ED visits within a 6-month period. Well education and poor general health resulted in an increase in the rate of SCD-related ED visits. These findings can contribute to medical practitioners’ ability to manage and educate patients with SCD.

## Abbreviations

ED: Emergency department
HRQoL: Health-related quality of life
QoL: Quality of life
SCD: Sickle cell disease
SF-36: 36-Item Short Form Health Survey
